# On non-BPS effective actions of string theory

**DOI:** 10.1140/epjc/s10052-018-5868-z

**Published:** 2018-05-17

**Authors:** Ehsan Hatefi

**Affiliations:** 1 Institute for Theoretical Physics, TU Wien, Wiedner Hauptstrasse 8-10/136, 1040 Vienna, Austria; 2 Faculty of Physics, University of Warsaw, ul. Pasteura 5, 02-093 Warsaw, Poland; 3 Faculty of Mathematics, Mathematical Institute, Charles University, 18675 Prague, Czech Republic

## Abstract

We discuss some physical prospective of the non-BPS effective actions of type IIA and IIB superstring theories. By dealing with all complete three and four point functions, including a closed Ramond–Ramond string (in terms of both its field strength and its potential), gauge (scalar) fields as well as a real tachyon and under symmetry structures, we find various restricted world volume and bulk Bianchi identities. The complete forms of the non-BPS scattering amplitudes including their Chan–Paton factors are elaborated. All the singularity structures of the non-BPS amplitudes, their all order $\alpha '$ higher-derivative corrections, their contact terms and various modified Bianchi identities are derived. Finally, we show that scattering amplitudes computed in different super-ghost pictures are compatible when suitable Bianchi identities are imposed on the Ramond–Ramond fields. Moreover, we argue that the higher-derivative expansion in powers of the momenta of the tachyon is universal.

## Introduction

D-branes have been realized to be the sources for Ramond–Ramond (RR) fields [1, 2]. RR couplings played important contributions to string theory. For instance to observe some of the application of RR couplings, one may consider the dissolving branes [3], K-theory and the Myers effect [4–6]. The other applications to RR couplings are related to the $N^3$ phenomena for M5-branes, dS solutions, entropy growth and geometrical applications to the effective actions [7–9].

The spectrum of the so-called non-BPS (unstable) branes includes massless states, tachyons, and an infinite number of massive states. There must be an Effective Field theory (EFT) for non-BPS branes where one integrates out all the massive states and hence the spectrum involves just the tachyon and massless states [10]. We will not point out cosmological applications for unstable branes. On general grounds, one might expect that D-branes and SD-branes have similar effective actions. The effective action of these branes has to have two parts. It consists of the extensions of the usual DBI and Wess–Zumino (WZ) actions where the tachyon mode is embedded into these effective actions. By applying the conformal field theory (CFT) methods [11], the leading order effective couplings of the fermions with tachyons were found in [12, 13] as

$$\begin{aligned} S=-T_pV(T)\sqrt{-\det (\eta _{ab}+2\pi \alpha 'F_{ab}-2\pi \alpha '\bar{\Psi }\gamma _b\partial _a\Psi +\pi ^2\alpha '^2\bar{\Psi }\gamma ^{\mu }\partial _a\Psi \bar{\Psi }\gamma _{\mu }\partial _b\Psi + 2\pi \alpha ' D_{a}T D_{b}T )}. \end{aligned}$$

In the above action, $F_{ab}$ is the field strength of the gauge field, $\bar{\Psi }\gamma _{\mu }\partial ^{\mu }\Psi$ is the kinetic term of fermion fields, *DT* is the covariant derivative of the tachyon $(D_aT=\partial _a T-i[A_a,T])$. On the other hand the Chern–Simons action for BPS branes was constructed in [14]. Using Boundary String Field Theory (BSFT), one has the tachyon’s kinetic term in the DBI part [15] as follows:

1 $$\begin{aligned} S_\mathrm{DBI}\sim & {} \int \mathrm{d}^{p+1}\sigma \, e^{-2\pi T^2} F(2\pi \alpha 'D^aTD_aT),\nonumber \\ F(x)= & {} \frac{4^xx\Gamma (x)^2}{2\Gamma (2x)}. \end{aligned}$$

The WZ action in BSFT approach is found to be

2 $$\begin{aligned} S_\mathrm{WZ}= & {} \mu _p' \int _{\Sigma _{(p+1)}} C \wedge \mathrm{Str}\,e^{i2\pi \alpha '\mathcal {F}}, \end{aligned}$$

where $C_{(p+1)}$ is the RR potential $(p+1)$ form-field and the super-connection’s curvature would be given by

$$\begin{aligned} i{\mathcal {F}} = \left( \begin{array}{cc} iF -\beta '^2 T^2 &{} \beta ' DT \\ \beta ' DT &{} \quad iF -\beta '^2T^2 \end{array} \right) , \end{aligned}$$

$\beta '$ is the normalization constant and $\mu _p'$ is the RR charge of the brane.

If we expand the exponential in (2), then we obtain various couplings as follows:

3 $$\begin{aligned} S_\mathrm{WZ}= & {} 2\beta '\mu _p' (2\pi \alpha ')\mathrm{Tr}\,\bigg (C_{p}\wedge DT + (2\pi \alpha ')C_{p-2}\wedge DT\wedge F\nonumber \\&+\, \frac{(2\pi \alpha ')^2}{2}C_{p-4}\wedge F\wedge F\wedge DT\bigg ) . \end{aligned}$$

For the sake of the higher-derivative corrections, we work with the second approach of exploring effective actions, which is the scattering amplitude formalism. In this approach the tachyon’s kinetic term is embedded into the DBI action as follows:

4 $$\begin{aligned}&S_\mathrm{DBI}\sim \int \mathrm{d}^{p+1}\sigma \mathrm{STr}\,\left( \frac{}{}V({ T^iT^i})\sqrt{1+\frac{1}{2}[T^i,T^j][T^j,T^i])}\right. \nonumber \\&\quad \left. \times \sqrt{-\det (\eta _{ab} +2\pi \alpha 'F_{ab}+2\pi \alpha 'D_a{ T^i}(Q^{-1})^{ij}D_b{ T^j})} \right) \,,\nonumber \\ \end{aligned}$$

where $V({T^iT^i})=e^{-\pi { T^iT^i}/2}$, $Q^{ij}=I\delta ^{ij}-i[T^i,T^j], T^1=T\sigma _1, T^2=T\sigma _2$ and $\sigma _1,\sigma _2$ are Pauli matrices. The trace in (4) should be symmetric for all $F_{ab},D_a{ T^i}$, $[T^i,T^j]$ matrices. If all Chan–Paton factors are taken into account, then this action would produce consistent results with all momentum expansions of three and four point functions of a closed string RR field and either the two, three tachyon or the two tachyon two gauge/scalar field amplitude.

On the stable point, the tachyon potential and its effective action get replaced by the well-known tachyon DBI action [16, 17] with potential $T^4V(T^2)$. The WZ part of the action in this approach has the same formula as appearing in (2). Using the S-matrix method the normalization constants of $\beta ',\beta$ for the non-BPS and brane–antibrane system are discovered to be $\beta '=\frac{1}{\pi }\sqrt{\frac{6\ln (2)}{\alpha '}}$ and $\beta =\frac{1}{\pi }\sqrt{\frac{2\ln (2)}{\alpha '}}$ [18]. It is worth mentioning that the super-connection’s structure for the WZ action was found by the S-matrix approach in [19].

The aim of the paper is to show that the scattering amplitudes computed in different super-ghost pictures are compatible when suitable Bianchi identities are imposed on the RR fields. Moreover, we argue that the higher-derivative expansion in powers of the momenta of the tachyon is universal.

The outline of this paper is as follows. First we find all three point functions including a gauge field, a tachyon and a closed string RR in all asymmetric and symmetric pictures of the closed string RR. By doing so, not only do we find some restricted Bianchi identities on both world volume and transverse directions of non-BPS branes, but also we explore all their infinite higher-derivative corrections. It is believed that due to a supersymmetry transformation BPS the S-matrices do not generate a Bianchi identity. To get consistent results for four point functions of the two gauge fields, a tachyon and a closed string RR field in their asymmetric and symmetric pictures, we discover various restricted Bianchi identities. Eventually we have to do with a universal expansion for tachyon and construct all different singularity structures of $\langle V_{C^{-2}} V_{A^{0}} V_{A^{0}} V_{T^{0}} \rangle$ as well as all order $\alpha '$ higher-derivative corrections to the various couplings of the type IIA, IIB superstring theories.

## All order $\langle V_{C^{-2}} V_{A^{0}} V_{T^{0}} \rangle$

In this section we would like to apply CFT methods to derive the complete S-matrix elements of a closed string RR, a gauge field and a tachyon. The total super-ghost charge for disk level amplitude must be $-\,2$. First we choose an asymmetric closed string RR field (which carries total $-\,2$ super-ghost charge) and hence the gauge field and a tachyon must be put in zero picture. This S-matrix can be obtained if one finds the correlation functions of the following vertex operators:5It is argued in [20] that the vertices of a non-BPS D-brane need to carry internal degrees of freedom or a Chan–Paton (CP) matrix. This is because if we set the tachyon to zero, then the WZ effective action of non-BPS branes gets reduced to the WZ action of BPS branes. Hence, we impose an identity internal CP matrix to all massless fields including gauge (scalar) and RR fields in zero picture. It is discussed in [21] that a Picture Changing Operator (PCO) carries a CP matrix $\sigma _3$. It is explained in [22] that the tachyon in zero and the ($-1$) picture carries $\sigma _1$ and $\sigma _2$ CP factors. $\langle V_{C^{-1}} V_{T^{-1}}\rangle$ makes sense in the world volume of non-BPS branes. This fixes the CP factor of RR in the $(-1/2, -1/2)$ picture to be $\sigma _3\sigma _1$. By applying PCO to RR in the ($-1$) picture, we derive its CP factor in the ($-2$) picture to be $\sigma _1$ and the CP factor for the gauge field in the ($-1$) picture to be $\sigma _3$ where $\lambda$ is the external CP matrix for the U(N) gauge group.

We are looking for the disk level amplitude. The closed string will be located in the middle of the disk whereas all open strings are located at the boundary of the disk. The on-shell conditions are

$$\begin{aligned} q^2=p^2=0, \quad k_{2}^2=1/4 , q \cdot \xi _1=0. \end{aligned}$$

The definitions of the RR’s field strength and projection operator areFor type IIA (IIB) $n=2,4$, $a_n=i$ ($n=1,3,5$, $a_n=1$) and in spinor notationWe apply the doubling trick so that all the holomorphic parts of the fields can be used. Thus the following change of variables works:

$$\begin{aligned}&\tilde{X}^{\mu }(\bar{z}) \rightarrow D^{\mu }_{\nu }X^{\nu }(\bar{z}) \ , \ \ \ \tilde{\psi }^{\mu }(\bar{z}) \rightarrow D^{\mu }_{\nu }\psi ^{\nu }(\bar{z}) ,\nonumber \\&\quad \ \ \ \tilde{\phi }(\bar{z}) \rightarrow \phi (\bar{z})\,, \ \ \ \text{ and }\ \ \ \tilde{S}_{\alpha }(\bar{z}) \rightarrow M_{\alpha }{}^{\beta }{S}_{\beta }(\bar{z}) , \end{aligned}$$

with

$$\begin{aligned} D= & {} \left( \begin{array}{cc} -1_{9-p} &{} 0 \\ 0 &{} \quad 1_{p+1} \end{array} \right) ,\,\, \ \ \ \text{ and }\ \ \ \\ M_p= & {} \left\{ \begin{array}{cc}\frac{\pm i}{(p+1)!}\gamma ^{i_{1}}\gamma ^{i_{2}}\ldots \gamma ^{i_{p+1}} \epsilon _{i_{1}\ldots i_{p+1}}\,\,\,\,\mathrm{for}\, p \,\mathrm{even,}\\ \frac{\pm 1}{(p+1)!}\gamma ^{i_{1}}\gamma ^{i_{2}}\ldots \gamma ^{i_{p+1}}\gamma _{11} \epsilon _{i_{1}\ldots i_{p+1}} \,\,\,\,\mathrm{for}\, p \,\mathrm{odd.}\end{array}\right. \end{aligned}$$

Now one can use the following two-point functions for $X^{\mu },\psi ^\mu , \phi$:

6 $$\begin{aligned} \begin{aligned} \langle X^{\mu }(z)X^{\nu }(w)\rangle&= -\frac{\alpha '}{2}\eta ^{\mu \nu }\log (z-w), \\ \langle \psi ^{\mu }(z)\psi ^{\nu }(w) \rangle&= -\frac{\alpha '}{2}\eta ^{\mu \nu }(z-w)^{-1},\\ \langle \phi (z)\phi (w)\rangle&= -\log (z-w) . \end{aligned} \end{aligned}$$

The amplitude in an asymmetric picture is given bywhere $x_4=z=x+iy,x_5=\bar{z}=x-iy$ and

7 $$\begin{aligned} I_1= & {} ik_{2}^{a}\bigg (\frac{x_{42}}{x_{12}x_{14}}+\frac{x_{52}}{x_{12}x_{15}}\bigg ) 2^{-1/2}(x_{24}x_{25})^{-1/2}\nonumber \\&\times (x_{45})^{-3/4}(\gamma ^{b} C^{-1})_{\alpha \beta }. \end{aligned}$$

One uses the Wick-like rule [23] to obtain the correlation function for $I_2$,

$$\begin{aligned} I_2 = 2ik_{1c}\langle :S_{\alpha }(x_4): S_{\beta }(x_5):\psi ^{c}\psi ^{a}(x_1):\psi ^{b}(x_2):\rangle \end{aligned}$$

as follows:

$$\begin{aligned} I_2= & {} \bigg ((\Gamma ^{bac} C^{-1})_{\alpha \beta }+\frac{2Re[x_{14}x_{25}]}{x_{12}x_{45}}\bigg (\eta ^{bc}(\gamma ^{a} C^{-1})_{\alpha \beta }-\eta ^{ab}(\gamma ^{c} C^{-1})_{\alpha \beta }\bigg )\bigg )\\&\times \, 2ik_{1c} 2^{-3/2}(x_{14}x_{15})^{-1}(x_{24} x_{25})^{-1/2}(x_{45})^{1/4}. \end{aligned}$$

It can readily be shown that the amplitude is *SL*(2, *R*) invariant. We use the gauge fixing as $(x_1,x_2,z,\bar{z})=(x,-x,i,-i)$ and the Jacobian is $J=-2i(1+x^2)$. One reveals that $I_1$ has zero contribution to the S-matrix. Because the integrand is an odd function while the interval of the integral is symmetric.^1^ We introduce $t = -\frac{\alpha '}{2}(k_1+k_2)^2$ and $I_2$ is obtained byThe last two terms have just non-zero contributions to our amplitude. The final answer for the amplitude is8To be able to match the leading order of the S-matrix with the following coupling in the EFT:

9 $$\begin{aligned} 2i\beta '\mu '_p (2\pi \alpha ')^2\int _{\Sigma _{p+1}} \mathrm{Tr}\,(C_{p-2}\wedge F\wedge DT), \end{aligned}$$

we use $(\pi \beta '\mu _p'/2)$ as the normalization constant. $\beta '$ and $\mu _p'$ are known to be the WZ normalization constant and the RR brane’s charge. On the other hand, the result in symmetric cases (RR is written in the ($-1$) picture) for ${\mathcal {A}}^{A^0,T^{-1},C^{-1}}$ can be derived as10Accordingly ${\mathcal {A}}^{A^{-1},T^{0},C^{-1}}$ is found to be11By applying momentum conservation $(k_1+k_2+p)^a=0$ and making comparisons between (10) and (11), one obtains the following Bianchi identities:

12 $$\begin{aligned} p_b H_{a_0\ldots a_{p-2}}\epsilon ^{a_0\ldots a_{p-2}ba}=p_a H_{a_0\ldots a_{p-2}} \epsilon ^{a_0\ldots a_{p-2}ba}=0. \nonumber \\ \end{aligned}$$

All three point functions of a closed string RR, a tachyon and a scalar field in all symmetric and asymmetric pictures of RR can also be computed. The result for ${\mathcal {A}}^{\phi ^0,T^{-1},C^{-1}}$ is given byTo get a consistent result for the S-matrix, in the presence of all different pictures of closed string RR, the restricted Bianchi identity (12) must get replaced by the following Bianchi identity:

13 $$\begin{aligned} p^i \epsilon ^{a_0\ldots a_{p}}H_{a_0\ldots a_{p}}+p^a \epsilon ^{a_0\ldots a_{p-1}a}H^{i}_{a_0\ldots a_{p-1}}=0 . \end{aligned}$$

This modified Bianchi identity holds for all world volume and transverse directions of branes. The trace below is non-zero for the $p+1= n+2$ case,The trace that has the $\gamma ^{11}$ part indicates that the following relation holds:

$$\begin{aligned} p>3 , H_n=*H_{10-n} , \quad n\ge 5. \end{aligned}$$

Neither there are massless poles nor tachyon poles for this three point function. It is argued in [24] that the expansion of the non-BPS amplitudes in the presence of a closed string RR field makes sense if one applies the following constraint:

14 $$\begin{aligned} t=-p^ap_a\rightarrow \frac{-1}{4}. \end{aligned}$$

For the brane–antibrane configuration the above constraint gets replaced by $p^a p_a\rightarrow 0$ [25]. Hence, the precise momentum expansion for *CAT* is $t\rightarrow -1/4$. The expansion for the gamma function is

$$\begin{aligned} \sqrt{\pi }\frac{\Gamma [-t+1/4]}{\Gamma [3/4-t]}= & {} \pi \sum _{n=-1}^{\infty }c_n(t+1/4)^{n+1} , \end{aligned}$$

with the following coefficients:

$$\begin{aligned} c_{-1}= & {} 1, \quad c_0=2ln(2), \quad c_1=\frac{1}{6}(\pi ^2+12ln(2)^2),\ldots \end{aligned}$$

An infinite number of higher-derivative corrections to a $C_{p-2}$, a tachyon and a gauge field can be found by producing the contact terms in an EFT as follows:

15 $$\begin{aligned}&\frac{2i\beta '\mu _p'}{(p-2)!}(2\pi \alpha ')^2 C_{p-2}\nonumber \\&\quad \wedge \mathrm{Tr}\,\left( \sum _{n=-1}^{\infty }c_n(\alpha ')^{n+1} D_{a_1}\cdots D_{a_{n+1}}F \wedge D^{a_1}\ldots D^{a_{n+1}} DT\right) . \nonumber \\ \end{aligned}$$

Let us deal with the complete amplitude $\langle V_{C^{-2}} V_{A^0} V_{A^0} V_{T^0}\rangle$, to see what kinds of restricted Bianchi identities can be explored and also to see whether or not there are bulk singularity structures.

## The complete $\langle V_{C^{-2}} V_{A^0} V_{A^0} V_{T^0}\rangle$ amplitude

In order to find the complete form of the scattering amplitude of a tachyon, a potential RR $(p+1)$ form-field and two gauge fields $\langle V_{C^{-2}} V_{A^0} V_{A^0} V_{T^0}\rangle$, one needs to employ all CFT techniques. To achieve all singularities and contact interactions, we use the vertex operators. Note that, as clarified in [26], the CP factor of RR for the brane–antibrane system is different from the CP factor of non-BPS branes. RR vertex operators are introduced in [27]. One might refer for some of the BPS and non-BPS scattering amplitudes to [28–33].

Recently, an analysis of $\langle V_{C^{-2}} V_{\phi ^0} V_{\phi ^0}V_{T^0}\rangle$ was done; however, one cannot derive the result for $\langle V_{C^{-2}} V_{A^0} V_{A^0} V_{T^0}\rangle$ from it. This is because not all world volume couplings nor bulk terms have any effect in our new effective action. Given the presence of the tachyon, we cannot compare them with BPS branes’s effective action [34–38]. The closed form of the correlation functions is written down by the following^2^ and all the other kinematical relations can be found in [26]:16where

$$\begin{aligned} I= & {} |x_{12}|^{\alpha '^2 k_1\cdot k_2}|x_{13}|^{\alpha '^2 k_1\cdot k_3}|x_{14}x_{15}|^{\frac{\alpha '^2}{2} k_1\cdot p}|x_{23}|^{\alpha '^2 k_2\cdot k_3}|\nonumber \\&\times x_{24}x_{25}|^{\frac{\alpha '^2}{2} k_2\cdot p} |x_{34}x_{35}|^{\frac{\alpha '^2}{2} k_3\cdot p}|x_{45}|^{\frac{\alpha '^2}{4}p\cdot D\cdot p},\\ a^a_1= & {} ik_{2}^{a}\bigg (\frac{x_{42}}{x_{14}x_{12}}+\frac{x_{52}}{x_{15}x_{12}}\bigg )+ik_{3}^{a}\bigg (\frac{x_{43}}{x_{14}x_{13}}+\frac{x_{53}}{x_{15}x_{13}}\bigg ),\\ a^b_2= & {} -ik_{1}^{b}\bigg (\frac{x_{14}}{x_{42}x_{12}}+\frac{x_{15}}{x_{52}x_{12}}\bigg )-ik_{3}^{b}\bigg (\frac{x_{43}}{x_{42}x_{23}}+\frac{x_{53}}{x_{52}x_{23}}\bigg ),\\ a^{c}_{1}= & {} 2^{-1/2}x_{45}^{-3/4}(x_{34}x_{35})^{-1/2} (\gamma ^{c}C^{-1})_{\alpha \beta } ,\\ a^{cbd}_{2}= & {} 2^{-3/2}x_{45}^{1/4}(x_{34}x_{35})^{-1/2}(x_{24}x_{25})^{-1}\nonumber \\&\times \bigg \{(\Gamma ^{cbd}C^{-1})_{\alpha \beta }+\alpha ' h_1 \frac{Re[x_{24}x_{35}]}{x_{23}x_{45}}\bigg \},\\ a^{cae}_{3}= & {} 2^{-3/2}x_{45}^{1/4}(x_{34}x_{35})^{-1/2}(x_{14}x_{15})^{-1}\nonumber \\&\times \bigg \{(\Gamma ^{cae}C^{-1})_{\alpha \beta }+\alpha ' h_2 \frac{Re[x_{14}x_{35}]}{x_{13}x_{45}}\bigg \},\\ h_1= & {} \eta ^{dc} (\gamma ^{b}C^{-1})_{\alpha \beta }-\eta ^{bc} (\gamma ^{d}C^{-1})_{\alpha \beta },\\ h_2= & {} \eta ^{ec} (\gamma ^{a}C^{-1})_{\alpha \beta }-\eta ^{ac} (\gamma ^{e}C^{-1})_{\alpha \beta }. \end{aligned}$$

The last fermionic correlator $a_4^{cbdae}=<:S_{\alpha }(x_4): S_{\beta }(x_5):\psi ^{e}\psi ^{a}(x_1):\psi ^{d}\psi ^{b}(x_2):\psi ^{c}(x_3):>$ can be explored as follows:

17 $$\begin{aligned} a_4^{cbdae}= & {} \bigg \{(\Gamma ^{cbdae}C^{-1})_{{\alpha \beta }}+\alpha ' h_3\frac{Re[x_{14}x_{25}]}{x_{12}x_{45}}\nonumber \\&+\,\alpha ' h_4\frac{Re[x_{14}x_{35}]}{x_{13}x_{45}}+\,\alpha ' h_5 \frac{Re[x_{24}x_{35}]}{x_{23}x_{45}}\nonumber \\&+\, \alpha '^2h_6\bigg (\frac{Re[x_{14}x_{35}]}{x_{13}x_{45}}\bigg )\bigg (\frac{Re[x_{14}x_{25}]}{x_{12}x_{45}}\bigg )\nonumber \\&+\,\alpha '^2 h_7\bigg (\frac{Re[x_{14}x_{25}]}{x_{12}x_{45}}\bigg )^{2} \nonumber \\&+\,\alpha '^2 h_8 \bigg (\frac{Re[x_{14}x_{25}]}{x_{12}x_{45}}\bigg )\bigg (\frac{Re[x_{24}x_{35}]}{x_{23}x_{45}}\bigg ) \bigg \}\nonumber \\&\quad \times 2^{-5/2}x_{45}^{5/4}(x_{14}x_{15}x_{24}x_{25})^{-1}(x_{34}x_{35})^{-1/2},\nonumber \\ h_3= & {} \bigg (\eta ^{ed}(\Gamma ^{cba}C^{-1})_{\alpha \beta } -\,\eta ^{eb}(\Gamma ^{cda}C^{-1})_{\alpha \beta }\nonumber \\&- \eta ^{ad}(\Gamma ^{cbe}C^{-1})_{\alpha \beta } +\eta ^{ab}(\Gamma ^{cde}C^{-1})_{\alpha \beta } \bigg ),\nonumber \\ h_4= & {} \bigg (\eta ^{ec}(\Gamma ^{bda}C^{-1})_{\alpha \beta } -\eta ^{ac}(\Gamma ^{bde}C^{-1})_{\alpha \beta } \bigg ),\nonumber \\ h_5= & {} \bigg (\eta ^{dc}(\Gamma ^{bae}C^{-1})_{\alpha \beta } -\eta ^{bc}(\Gamma ^{dae}C^{-1})_{\alpha \beta }\bigg ),\nonumber \\ h_6= & {} \bigg (\eta ^{ed}\eta ^{ac}(\gamma ^{b}C^{-1})_{\alpha \beta }- \eta ^{eb}\eta ^{ac}(\gamma ^{d}C^{-1})_{\alpha \beta }\nonumber \\&- \,\eta ^{ec}\eta ^{ad}(\gamma ^{b}C^{-1})_{\alpha \beta }+ \eta ^{ec}\eta ^{ab}(\gamma ^{d}C^{-1})_{\alpha \beta }\bigg ),\nonumber \\ h_7= & {} \bigg (- \eta ^{ed}\eta ^{ab}(\gamma ^{c}C^{-1})_{\alpha \beta }+ \eta ^{eb}\eta ^{ad}(\gamma ^{c}C^{-1})_{\alpha \beta }\bigg ),\nonumber \\ h_8= & {} \bigg (-\eta ^{ed}\eta ^{bc}(\gamma ^{a}C^{-1})_{\alpha \beta }+\eta ^{eb}\eta ^{dc}(\gamma ^{a}C^{-1})_{\alpha \beta }\nonumber \\&+\,\eta ^{ad}\eta ^{bc}(\gamma ^{e}C^{-1})_{\alpha \beta }- \eta ^{ab}\eta ^{dc}(\gamma ^{e}C^{-1})_{\alpha \beta }\bigg ). \end{aligned}$$

We wrote all the S-matrix elements so that SL(2,R) invariance can be manifestly shown. By fixing three positions of the vertices, we can get rid of the volume of the Killing group. In order to get the algebraic answer for the amplitude, we fix the positions of open strings as

$$\begin{aligned} x_{1}= & {} 0 ,\quad x_{2}=1,\quad x_{3}\rightarrow \infty . \end{aligned}$$

Eventually one needs to take a 2D complex integrals on the location of the closed string RR on the upper half plane as follows:

18 $$\begin{aligned} \int \mathrm{d}^2 \!z |1-z|^{a} |z|^{b} (z - \bar{z})^{c} (z + \bar{z})^{d} \end{aligned}$$

where $d=0,1,2$ and *a*, *b*, *c* are written in terms of the following Mandelstam variables:

$$\begin{aligned} s= & {} \frac{-\alpha '}{2}(k_1+k_3)^2, \quad t=\frac{-\alpha '}{2}(k_1+k_2)^2,\\ u= & {} \frac{-\alpha '}{2}(k_2+k_3)^2, \quad s'=s+\frac{1}{4}, \quad u'=u+\frac{1}{4}. \end{aligned}$$

For $d=0,1$ and $d=2,3$ the algebraic solutions for the integrals are obtained in [39] and [40], respectively. The final form of the amplitude is

19 $$\begin{aligned} {\mathcal {A}'}^{C^{-2} A^0 A^{0} T^0}= & {} {\mathcal {A}'}_{1}+{\mathcal {A}'}_{2} +{\mathcal {A}'}_{3} \end{aligned}$$

where20The functions $L_1,L_2,L_3$ are

$$\begin{aligned}&L_1=(2)^{-2(t+s+u)-1}\pi \\&\quad \times {\frac{\Gamma (-u+\frac{3}{4}) \Gamma (-s+\frac{3}{4})\Gamma (-t)\Gamma (-t-s-u)}{\Gamma (-u-t+\frac{3}{4})\Gamma (-t-s+\frac{3}{4})\Gamma (-s-u+\frac{1}{2})}} , \\&L_2=(2)^{-2(t+s+u)-1}\pi \nonumber \\&\quad \times {\frac{\Gamma (-u+\frac{3}{4}) \Gamma (-s-\frac{1}{4})\Gamma (-t+1)\Gamma (-t-s-u)}{\Gamma (-u-t+\frac{3}{4})\Gamma (-t-s+\frac{3}{4})\Gamma (-s-u+\frac{1}{2})}} , \\&L_3=(2)^{-2(t+s+u)}\pi \\&\times {\frac{\Gamma (-u+\frac{1}{4}) \Gamma (-s+\frac{1}{4})\Gamma (-t+\frac{1}{2})\Gamma (-t-s-u-\frac{1}{2})}{\Gamma (-u-t+\frac{3}{4}) \Gamma (-t-s+\frac{3}{4})\Gamma (-s-u+\frac{1}{2})}}. \end{aligned}$$

This amplitude satisfies Ward identities related to both gauge fields. We expand the amplitude in such a way that all tachyon and massless poles can be obtained from the EFT. Finally, we produce all contact interactions to all orders in $\alpha '$. One thinks that the amplitude in the asymmetric case has non-zero terms for the $p+1=n$ case; however, these terms are not gauge invariant. These terms are

21 $$\begin{aligned}&2^{1/2}i \mathrm{Tr}\,(P_{-}C\!\!\!\!/\,_{(n-1)}M_p\gamma ^{c})\bigg [ 2(k_2\cdot \xi _1)(k_1\cdot \xi _2) k_{3c} \nonumber \\&\quad \times \bigg (-4M_5+4K_2+M_9 -4\left( \frac{1}{4}M_9+K_2-M_5\right) \bigg )\nonumber \\&\quad +\xi _{2c}k_2\cdot \xi _1\bigg (4u'\bigg (-M_4+\frac{1}{2}M_5+\frac{1}{2}M_{11}-\frac{1}{4}M_9\bigg )\nonumber \\&\quad -4s'\bigg (\frac{1}{4}M_9-\frac{1}{2}M_{5}\bigg )\bigg ) +\xi _{2c}k_3\cdot \xi _1\bigg (-2u'\bigg (-M_{11}+\frac{1}{2}M_9\bigg )\nonumber \\&\quad +4t\bigg (\frac{1}{4}M_9-\frac{1}{2}M_{5}\bigg )\bigg )+\xi _{1c}k_3\cdot \xi _2\bigg (2s'M_{11}-s'M_9\nonumber \\&\quad -4t\bigg (\frac{1}{4}M_9-\frac{1}{2}M_{11}+M_4-\frac{1}{2}M_{5}\bigg )\bigg ) +\xi _{1c}k_1\nonumber \\&\quad \cdot \xi _2\bigg (-2s'M_{5}+s'M_9 +4u'\bigg (\frac{1}{4}M_9-\frac{1}{2}M_{11}+M_4-\frac{1}{2}M_{5}\bigg )\bigg )\bigg ].\nonumber \\ \end{aligned}$$

The sum of all coefficients of all terms in parentheses of (21) is zero. This means that they disappear from the ultimate form of the amplitude. All $K_2, M$ functions are written in terms of gamma functions and for the sake of this paper we will not mention their forms. This confirms that there is no bulk singularity term for this S-matrix.

## The complete $\langle V_{C^{-1}} V_{A^{0}} V_{A^{-1}} V_{T^{0}}\rangle$ amplitude

This S-matrix in terms of the field strength of the closed string RR field, that is, $\langle V_{C^{-1}} V_{A^0} V_{A^{-1}}V_{T^0}\rangle$ has not been calculated yet. Using CFT, we explore the amplitude of $\langle V_{C^{-1}} V_{A^0} V_{A^{-1}}V_{T^0}\rangle$. It can be found by the following correlations:22

23 $$\begin{aligned} I= & {} |x_{12}|^{4k_1\cdot k_2}|x_{13}|^{4k_1\cdot k_3}|x_{14}x_{15}|^{2k_1\cdot p}|x_{23}|^{4k_2\cdot k_3}\nonumber \\&\times |x_{24}x_{25}|^{2k_2\cdot p} |x_{34}x_{35}|^{2k_3\cdot p}|x_{45}|^{p\cdot D\cdot p}\nonumber \\ a^a_1= & {} ik_2^{a}\bigg (\frac{x_{42}}{x_{14}x_{12}}+\frac{x_{52}}{x_{15}x_{12}}\bigg )\nonumber \\&+\,ik_3^{a}\bigg (\frac{x_{43}}{x_{14}x_{13}}+\frac{x_{53}}{x_{15}x_{13}}\bigg ). \end{aligned}$$

One needs to know the following correlation functions:

24 $$\begin{aligned} a^{cb}_2= & {} \langle :S_{\alpha }(x_4): S_{\beta }(x_5):\psi ^{b}(x_2):\psi ^{c}(x_3):\rangle \nonumber \\= & {} \bigg \{(\Gamma ^{cb}C^{-1})_{\alpha \beta }-2\eta ^{bc} \frac{Re[x_{24}x_{35}]}{x_{23}x_{45}}\bigg \}\nonumber \\&\times 2^{-1}(x_{24}x_{25}x_{34}x_{35})^{-1/2}x_{45}^{-1/4}. \end{aligned}$$

One obtains the correlation function of a current and two fermion fields in two different locations in the presence of two spin operator, that is, $I_2^{cbad}=\langle :S_{\alpha }(x_4): S_{\beta }(x_5):\psi ^{d}\psi ^{a}(x_1):\psi ^{b}(x_2):\psi ^{c}(x_3):\rangle$ as

$$\begin{aligned} I_2^{cbad}= & {} \bigg \{(\Gamma ^{cbad}C^{-1})_{{\alpha \beta }}+\alpha ' b_1\frac{Re[x_{14}x_{25}]}{x_{12}x_{45}}\\&+\, \alpha ' b_2\frac{Re[x_{24}x_{35}]}{x_{23}x_{45}}+\alpha ' b_3 \frac{Re[x_{14}x_{35}]}{x_{13}x_{45}}\\&+\,\alpha '^2b_4\bigg (\frac{Re[x_{14}x_{35}]}{x_{13}x_{45}}\bigg )\bigg (\frac{Re[x_{14}x_{25}]}{x_{12}x_{45}}\bigg ) \bigg \}\nonumber \\&\times 2^{-2}x_{45}^{3/4}(x_{34}x_{35}x_{24}x_{25})^{-1/2}(x_{14}x_{15})^{-1},\\ b_1= & {} \bigg (\eta ^{bd}(\Gamma ^{ca}C^{-1})_{\alpha \beta } -\eta ^{ab}(\Gamma ^{cd}C^{-1})_{\alpha \beta } \bigg ),\\ b_2= & {} \bigg (-\eta ^{bc}(\Gamma ^{ad}C^{-1})_{\alpha \beta } \bigg ),\\ b_3= & {} \bigg (-\eta ^{cd}(\Gamma ^{ba}C^{-1})_{\alpha \beta } +\eta ^{ac}(\Gamma ^{bd}C^{-1})_{\alpha \beta }\bigg ),\\ b_4= & {} \bigg (-\eta ^{ac}\eta ^{db}+\eta ^{ab}\eta ^{dc}\bigg )(C^{-1})_{\alpha \beta }. \end{aligned}$$

We fixed three positions of the open strings as $x_{1}=0 ,x_{2}=1,x_{3}\rightarrow \infty$, and one takes integration on the position of closed string RR. Having set the gauge fixing, one would find the complete form of the integrand for ${\mathcal {A}}^{C^{-1} A^0 A^{-1} T^0}$ as follows:25The final answer is given by

26 $$\begin{aligned} {\mathcal {A}''}^{C^{-1} A^0 A^{-1} T^0}= & {} {\mathcal {A}''}_{1}+ {\mathcal {A}''}_{2}+{\mathcal {A}''}_{3} \end{aligned}$$

where27On the other hand, this amplitude for the following picture $\langle V_{C^{-1}}V_{A^0} V_{A^0} V_{T^{-1}} \rangle$ was computed to be

28 $$\begin{aligned} {\mathcal {A}}^{C^{-1} A^0 A^0 T^{-1}}= & {} {\mathcal {A}}_{1} +{\mathcal {A}}_{2}+{\mathcal {A}}_{3} \end{aligned}$$

where29In the next section we address the tachyon’s momentum expansion to be able to expand our S-matrix and, finally, we generate its non-zero couplings.

## Tachyon’s momentum expansion

In [24] it is conjectured that the momentum expansion for the tachyon is universal. Given the momentum conservation for a closed string RR and a tachyon, one reveals that $k_a+p_a=0$, therefore $p^ap_a$ must be sent to the mass of the tachyon ($k^2=-m^2$). Hence, one understands the fact that

$$\begin{aligned} p^ap_a \rightarrow \frac{1}{4} \end{aligned}$$

and that is just possible for SD-branes or euclidean branes. This means that amplitude makes sense for non-BPS SD-branes [41]. The coupling of the two tachyons and a gauge field is non-zero, so to be able to produce all tachyon and massless poles of the EFT, we need to employ a unique expansion for all non-BPS branes. Two Mandelstam variables should be sent to the mass of the tachyon as follows:

30 $$\begin{aligned} s+t+u=-p^a p_a-\frac{1}{4}, \quad t\rightarrow 0, s\rightarrow -\frac{1}{4}, u\rightarrow -\frac{1}{4} . \nonumber \\ \end{aligned}$$

$(i\mu '_p\beta '\pi ^{1/2})$ is the normalization constant and the closed forms of the expansions are

31 $$\begin{aligned} L_1= & {} -\pi ^{3/2}\bigg (\frac{1}{t}\sum _{n=-1}^{\infty }b_n(u'+s')^{n+1}\nonumber \\&+\sum _{p,n,m=0}^{\infty }e_{p,n,m}t^{p}(s'u')^{n}(s'+u')^m\bigg ) ,\nonumber \\ L_2= & {} -\pi ^{3/2}\bigg (\frac{1}{s'}\sum _{n=-1}^{\infty }b_n(u'+t)^{n+1}\nonumber \\&+\,\sum _{p, n,m=0}^{\infty }e_{p,n,m}s'^{p}(tu')^{n}(t+u')^m\bigg ); \end{aligned}$$

thus some of the above coefficients are found.^3^ Having taken (31), we would understand that $L_1,L_2,L_3$ have *t*-channel gauge fields and $s', u',(s'+t+u')$ tachyonic singularities. We make comparisons of the singularity structures as well as all contact terms. We then reconstruct all singularities in EFT and derive the restricted world volume Bianchi identities for non-BPS branes.

## Singularities and restricted Bianchi identities

Let us first compare singularity structures between $\langle V_{C^{-2}} V_{A^0} V_{A^0}V_{T^{0}}\rangle$ and $\langle V_{C^{-1}} V_{A^0} V_{A^{-1}}V_{T^0}\rangle$ with (29). If we use momentum conservation $k_{1a}+k_{2a}+k_{3a}=-p_a$ and $pC=H$ to the complete ${\mathcal {A}'}_{3}$ of (19), then we are able to produce all $(s'+t+u')$ channel poles ${\mathcal {A}}_{3}$ of (29). The first term ${\mathcal {A}'}_{2}$ produces the seventh term ${\mathcal {A}}_{2}$ that has tachyon singularities. Replacing $k_{3c}=-(k_{1c}+k_{2c}+p_c)$ for the second term of ${\mathcal {A}'}_{2}$, we obtain

32 $$\begin{aligned}&- 2^{5/2}iL_1 \mathrm{Tr}\,(P_{-}C\!\!\!\!/\,_{(n-1)}M_p \Gamma ^{cde})\nonumber \\&\quad \times (k_{1c}+k_{2c}+p_c)k_{2d}k_{1e}\xi _1\cdot \xi _2. \end{aligned}$$

Equation (32) is symmetric under both $k_{1c},k_{2c}$ and is antisymmetric in terms $\epsilon ^{a_0\ldots a_{p-3}cde}$ inside the trace; therefore, $k_{1c},k_{2c}$ have no contribution to our coupling. Using $pC=H$ we derive the eighth term of ${\mathcal {A}}_{2}$ that has $(s'+t+u')$ tachyon singularities. The above arguments hold for the third term of ${\mathcal {A}'}_{2}$ so the third term of ${\mathcal {A}'}_{2}$ reconstructs the second term of ${\mathcal {A}}_{2}$ that has $s'$-channel tachyon poles. If we apply momentum conservation to the sixth term of ${\mathcal {A}'}_{2}$, we find the following interaction:33$k_{1c}$ has no effect on the above interaction and using $pC=H$, (33) regenerates all $u'$ channel tachyon singularities (the fifth term of ${\mathcal {A}}_{2}$). Having applied momentum conservation to the fourth term ${\mathcal {A}'}_{2}$ we obtain34$k_{2c}$ has no contribution to the above interaction and using $pC=H$, one reproduces the first term of ${\mathcal {A}}_{2}$. Now adding the contribution $k_{1c}$ of (34) to the fifth term of ${\mathcal {A}'}_{2}$ we obtain35which is the sixth term of ${\mathcal {A}}_{2}$. By applying momentum conservation to the seventh term ${\mathcal {A}'}_{2}$ we get36$k_{2c}$ has no contribution; taking $pC=H$, we generate the third term ${\mathcal {A}}_{2}$ of (29). One might suppose that $k_{1c}$ from (36) is an extra singularity; however, the presence of this term is needed. Indeed if we take the contribution $k_{1c}$ from (36) and add it to the last term of ${\mathcal {A}'}_{2}$ we find37which is exactly the third term ${\mathcal {A}}_{2}$. Therefore, we are able to produce not only all t-channel singularities (29) but also all its $s',u',(t+s'+u')$ channel tachyon singularities of $\langle V_{C^{-2}} V_{A^0} V_{A^0}V_{T^{0}}\rangle$ are produced. Let us deal with singularities that appear in (27). ${\mathcal {A}''}_{3}$ is the same as ${\mathcal {A}}_{3}$. The fourth and fifth terms of ${\mathcal {A}''}_{2}$ are equivalent to the fifth and seventh terms of ${\mathcal {A}}_{2}$. Applying momentum conservation to the sixth term ${\mathcal {A}''}_{2}$, we get38$k_{1c}$ has no contribution to the above interaction. The contribution from $k_{2c}$ produces the eighth term ${\mathcal {A}}_{2}$, and to make a consistent result for both symmetric amplitudes, one imposes the following restricted Bianchi identity:

39 $$\begin{aligned} p_c H_{a_{0}\ldots a_{p-2}} \epsilon ^{a_{0}\ldots a_{p-2}cd}=0. \end{aligned}$$

Using the direct scattering amplitude $\langle V_{C^{-2}} V_{\phi ^{0}} V_{\phi ^0} V_{T^{0}}\rangle$ the following Bianchi identity holds in terms of both the RR field strength and the RR potential in the complete space-time:

40 $$\begin{aligned}&\epsilon ^{a_0\cdots a_{p}} \bigg ( -p_{a_{p}} (p+1) H^{ij}_{a_0\cdots a_{p-1}}-p^j H^{i}_{a_0\cdots a_{p}}+p^i H^{j}_{a_0\cdots a_{p}}\bigg )\nonumber \\&\quad =d H^{p+2}=0 \end{aligned}$$

or

41 $$\begin{aligned}&p_{a_{0}} \epsilon ^{a_0\cdots a_{p}} \bigg ( -p_{a_{p}} p (p+1) C_{ija_1\cdots a_{p-1}}-p^j C_{ia_1\cdots a_{p}}\nonumber \\&\quad +\,p^i C_{ja_1\cdots a_{p}}\bigg )=0. \end{aligned}$$

If we apply momentum conservation to the first and third terms ${\mathcal {A}''}_{2}$ and simultaneously take into account the restricted world volume (39), then we actually reconstruct the sum of the first and sixth terms of ${\mathcal {A}}_{2}$ as well as the third and fourth terms ${\mathcal {A}}_{2}$. The same holds for the second term ${\mathcal {A}''}_{2}$ and we regenerate the second term ${\mathcal {A}}_{2}$. Hence, in comparison with (29) and using the restricted world volume Bianchi identities we are able to produce all t-channel gauge field singularities as well as $s',u',(t+s'+u')$ channel tachyon singularities of $\langle V_{C^{-1}} V_{A^0} V_{A^{-1}}V_{T^{0}}\rangle$. Unlike the $\langle V_{C^{-2}} V_{\phi ^0} V_{\phi ^{0}}V_{T^{0}}\rangle$ analysis, here we have no bulk singularity structures at all. Hence, brane singularities have been matched without producing any extra residual contact interactions.

## Contact term comparisons

To be able to obtain all the restricted Bianchi identities, we try to compare all contact interactions between (19) and (27) with all order contact terms of (29). If we replace $k_{3c}=-(k_{1c}+k_{2c}+p_c)$ to ${\mathcal {A}'}_{1}$ of (19) (also with ${\mathcal {A}''}_{1}$) and use the following Bianchi identity:

42 $$\begin{aligned} p_c \epsilon ^{a_0..a_{p-5}cbda}=0, \end{aligned}$$

then we produce all contact interactions of ${\mathcal {A}}_{1}$ for the $p=n+3$ case. The leading order couplings can be produced if we would normalize the amplitude by $(\mu '_p\beta '\pi ^{1/2})$ and compare it with the following coupling in the EFT:

43 $$\begin{aligned} \beta '\mu _p'(2\pi \alpha ')^{3} \mathrm{Tr}\,(C_{p-4}\wedge F\wedge F\wedge . DT) \end{aligned}$$

Recently the method of getting all order contact interactions has been released in [24, 26]. One can apply the higher-derivative corrections to the EFT couplings to produce all non-leading terms. For example, if we replace the expansion of $L_3$ in the amplitude, then one can derive all order contact interactions of the amplitude for the $p=n+3$ case as follows:

44 $$\begin{aligned}&8\beta '(\pi \alpha '^3)\mu _p'\bigg [\sum _{n=0}^{\infty }c_{n}\left( \frac{\alpha '}{2}\right) ^{n}(D^aD_a)^n\mathrm{Tr}\,(C_{p-4}\wedge F\wedge F\wedge DT)\nonumber \\&\quad +\sum _{p,n,m=0}^{\infty }f_{p,n,m}\left( \frac{\alpha '}{2}\right) ^{p}(D^aD_a)^p\left( \alpha '\right) ^{2m+n} C_{p-4}\wedge \mathrm{Tr}\,\nonumber \\&\quad \times \bigg ( D^{a_1}\cdots D^{a_{m}} D^{b_1}\cdots D^{b_{n}}((F\wedge D^{a_{m+1}}\cdots D^{a_{2m}}F)\wedge D_{b_1}\nonumber \\&\quad \cdots D_{b_{n}}D_{a_{1}}\cdots D_{a_{2m}}DT)\bigg )\bigg ] . \end{aligned}$$

Note that both ${\mathcal {A}''}_{1}$ and ${\mathcal {A}'}_{1}$ satisfy the Ward identity associated with the gauge fields.

Making use of the Bianchi identities we are able to generate all contact interactions $\langle V_{C^{-2}} V_{A^0} V_{A^0}V_{T^{0}}\rangle$ from (29) without any ambiguity. For instance, the first contact term of the amplitude for the $p=n+1$ case is

45 $$\begin{aligned}&\frac{32}{(p-1)!}(\mu '_p\beta '\pi ^{2})H_{a_{0}\cdots a_{p-2}}\xi _{1a_{p}}\xi _{2a_{p-1}}\epsilon ^{a_{0}\cdots a_{p}} . \end{aligned}$$

This contact interaction can be reconstructed by taking into account the following gauge invariant coupling in an EFT:

46 $$\begin{aligned} 2\beta '\mu _p'(2\pi \alpha ')^2\mathrm{Tr}\,(C_{p-2}\wedge F\wedge DT) . \end{aligned}$$

Notice that (43) is found by expanding the exponential of WZ action and using the multiplication rule of the super-matrices. If we consider the expansions of $L_1,L_2$ into the amplitude then one finds the contact interactions to the next leading order for the $p=n+1$ case

47 $$\begin{aligned}&\frac{32}{(p-1)!}(\mu '_p\beta '\pi ^{2})H_{a_{0}\cdots a_{p-2}}\epsilon ^{a_{0}\cdots a_{p}} \nonumber \\&\quad \times \bigg \{-\frac{\pi ^2}{6} \bigg (2k_2\cdot \xi _1 k_{2a_{p-1}}\xi _{2a_{p}} -2k_1\cdot \xi _2 k_{1a_{p-1}}\xi _{1a_{p}}\nonumber \\&\quad +\,2k_1\cdot \xi _2\xi _{1a_{p-1}}k_{2a_{p}}+2k_2\cdot \xi _1\xi _{2a_{p}}k_{1a_{p-1}}-t\xi _{1a_{p}}\xi _{2a_{p-1}}\nonumber \\&\quad +\,2\xi _1\cdot \xi _2k_{1a_{p}}k_{2a_{p-1}}\bigg )\bigg [t+2(s'+u')\bigg ]\nonumber \\&\quad +\,\frac{\pi ^2}{6}\xi _{1a_{p}}\xi _{2a_{p-1}}(s'+u')^{2}\nonumber \\&\quad +\,\bigg (\frac{\pi ^2}{3}k_3\cdot \xi _1k_{2a_{p-1}}\xi _{2a_{p}}\bigg [2(t+u')+s'\bigg ]-[1\leftrightarrow 2]\bigg )\bigg \} . \nonumber \\ \end{aligned}$$

All terms in (47) are related to the corrections of the EFT couplings. One can explore the following EFT couplings that regenerate the contact terms in (47):

48 $$\begin{aligned}&-\frac{1}{12}\beta '\mu _p'(2\pi \alpha ')^4\bigg [-iD^{\beta }F_{a\alpha }D^{\alpha }F_{b\beta }D_cT\nonumber \\&\quad +\,\frac{3i}{2}F_{ac}D_{\alpha }F_{\beta b}D^{\alpha }D^{\beta }T-\frac{3i}{2}D_{\alpha }F_{\beta b }F_{ac}D^{\alpha }D^{\beta }T\nonumber \\&\quad -\,\frac{1}{2}D_aD^{\alpha }D_cF_{b\alpha }D_{\beta }D^{\beta }T +F_{a\alpha }D^{\beta }D^{\alpha }D_{\beta }D_bD_cT\nonumber \\&\quad -\frac{1}{2}D_aD^{\alpha }D_{\beta }D^{\beta }F_{b\alpha }D_cT+D_bD_cF_{a\alpha }D^{\beta }D^{\alpha }D_{\beta }T\nonumber \\&\quad +\,4D^{\alpha }D_a D_cF_{\beta b}D_{\alpha }D^{\beta }T-\frac{1}{2}D_aF_{\alpha \beta }D_bD^{\alpha }D^{\beta }D_cT\nonumber \\&\quad -\,D_aD^{\beta }D_{\beta }D_cF_{b\alpha }D^{\alpha }T+2D_bD^{\alpha }D^{\beta }F_{a\alpha }D_{\beta }D_cT\nonumber \\&\quad +\,D^{\alpha }D_{\alpha }D_cF_{\beta b}D^{\beta }D_aT\nonumber \\&\quad +\,D_aD^{\beta }D_{\beta }F_{ b\alpha }D^{\alpha }D_cT+\frac{1}{2}D^{\beta }D^{\alpha }D_{\beta }D_cF_{a\alpha }D_b T\nonumber \\&\quad -\,\frac{1}{2}D^{\alpha }D^{\beta }F_{ab} D_{\alpha }D_{\beta }D_cT\bigg ] \frac{1}{(p-2)!}C_{a_{0}\cdots a_{p-3}}\epsilon ^{a_{0}\cdots a_{p-3}abc}\nonumber \\ \end{aligned}$$

where the covariant derivative of the tachyon is $D_aT=\partial _aT-i[A_a,T]$. Note that by the direct scattering amplitude of a closed string RR field, a tachyon and a gauge field in Sect. 2, we derived all order $\alpha '$ higher-derivative corrections to the last coupling of (48).

### All $(t+s'+u')$-channel tachyon singularities

Let us explore all $(t+s'+u')$- channel tachyon singularities of the amplitude ${\mathcal {A}'}_3$ for the $p+1=n$ case. Extracting the trace and normalizing the amplitude we derive them as follows:

49 $$\begin{aligned}&p_c C_{a_{0}\cdots a_{p-1}}\epsilon ^{a_{0}\cdots a_{p-1}c}\bigg (-t(k_3\cdot \xi _1)(k_3\cdot \xi _2)+(k_3\cdot \xi _2)(k_2\cdot \xi _1)s'\nonumber \\&\quad +\,(k_3\cdot \xi _1)(k_1\cdot \xi _2)u'+\frac{1}{2}(\xi _1\cdot \xi _2)s'u' \bigg )\nonumber \\&\quad \times \sum _{n,m=0}^{\infty }c_{n,m} (s'^{m}u'^{n}+s'^{n}u'^{m}) \frac{32 \beta '\mu '_p\pi ^3}{(s'+t+u')p!} , \end{aligned}$$

which satisfies the Ward identity. These poles can be constructed by employing a WZ coupling $2i\mu '_p\beta '(2\pi \alpha ')\int C_{p}\wedge DT$ and all order higher-derivative corrections to two tachyon–two gauge field couplings. In the effective field theory all singularities are derived by the following sub-amplitude and vertices:

50 $$\begin{aligned} \begin{aligned}{\mathcal {A}}&=V^{\alpha }(C_{p},T)G^{\alpha \beta }(T)V^{\beta }(T,T_3,A_1,A_2),\\ G^{\alpha \beta }(T)&=\frac{i\delta ^{\alpha \beta }}{(2\pi \alpha ') T_p (s'+u'+t)} , \\ V^{\alpha }(C_{p},T)&=2i\mu '_p\beta '(2\pi \alpha ')\frac{1}{p!}\epsilon ^{a_0\cdots a_{p}}C_{a_0\cdots a_{p-1}} k_{a_p} . \end{aligned} \end{aligned}$$

Replacing the vertex of two tachyon-two gauge field couplings in the above field theory amplitude, we obtain all tachyon singularities in the EFT as follows:

51 $$\begin{aligned}&32\pi \alpha '^{2}\beta '\mu _p'\frac{ \epsilon ^{a_{0}\cdots a_{p-1}c} p_c C_{a_{0}\cdots a_{p-1}}}{p!(s'+t+u')}\nonumber \\&\quad \times \sum _{n,m=0}^{\infty }\bigg ((a_{n,m}+b_{n,m})[s'^{m}u'^{n}+s'^{n}u'^{m}]\nonumber \\&\quad \times \bigg [-t(k_3\cdot \xi _2)(k_3\cdot \xi _1)+(k_2\cdot \xi _1)(k_3\cdot \xi _2)s'\nonumber \\&\quad +\,(k_1\cdot \xi _2)(k_3\cdot \xi _1)u'+(\xi _1\cdot \xi _2)\frac{1}{2}u's' \bigg ]\bigg ) . \end{aligned}$$

Some of the coefficients are

$$\begin{aligned}&a_{0,0}=-\frac{\pi ^2}{6},\quad b_{0,0}=-\frac{\pi ^2}{12},\quad a_{1,0}=2\zeta (3),\\&\,a_{0,1}=0,\quad b_{0,1}=b_{1,0}=-\zeta (3). \end{aligned}$$

These poles (51) are exactly the ones that appeared in S-matrix elements (49).

#### All $u',s'$ channel tachyon singularities

Given the symmetries of the amplitude, we reconstruct all $u'$-channel poles in the EFT. Like by exchanging momenta and polarizations, all $s'$-channel singularities can also be examined:

52 $$\begin{aligned}&\frac{32 \mu '_p\beta '\pi ^{2}}{(p-2)!} p_c C_{a_{0}\cdots a_{p-3}} \epsilon ^{a_{0}\cdots a_{p-3}cae}\nonumber \\&\quad \times \sum _{n=-1}^{\infty }b_n \frac{(s'+t)^{n+1}}{u'}(2k_3\cdot \xi _2)k_{1a}\xi _{1e} . \end{aligned}$$

All these $u'$-channel poles can be constructed by the following rule:

53 $$\begin{aligned} {\mathcal {A}}= & {} V^{\alpha }(C_{p-2},A_1,T)G^{\alpha \beta }(T)V^{\beta }(T,T_3,A_2) . \end{aligned}$$

$V^{\beta }(T,T_3,A_2)$ should be found from the non-Abelian kinetic term of the tachyons in DBI action. If we employ the corrections that we got from WZ coupling $2i\beta '\mu '_p \int C_{p-2}\wedge F\wedge DT$ in (15), then we obtain the higher order vertex of $V^{\alpha }(C_{p-2},A_1,T)$ and the other vertices as follows:

54 $$\begin{aligned}&V^{\beta }(T,T_3,A_2)=iT_p(2\pi \alpha ')(k_3-k)\cdot \xi _2,\nonumber \\&V^{\alpha }(C_{p-2},A_1,T) =2\mu '_p\beta '\frac{(2\pi \alpha ')^{2}}{(p-2)!}\epsilon ^{a_0\cdots a_{p-1}c} p_c \nonumber \\&\quad \times C_{a_0\cdots a_{p-3}}k_{1a_{p-2}}\xi _{1a_{p-1}} \sum _{n=-1}^{\infty }b_n(\alpha 'k_1\cdot k)^{n+1}. \end{aligned}$$

*k* is the off-shell tachyon momentum. Replacing (54) inside (53), we obtain all order $u'$ channel tachyon poles in an EFT:

$$\begin{aligned} {\mathcal {A}}= & {} \frac{2\mu '_p\beta '(2\pi \alpha ')^{2}}{(p-2)!u'}\epsilon ^{a_{0}\cdots a_{p-1}c} p_c C_{a_{0}\cdots a_{p-3}} k_{1a_{p-2}}\xi _{1a_{p-1}} (2k_3\cdot \xi _2)\nonumber \\&\times \sum _{n=-1}^{\infty }b_n (t+s')^{n+1}, \end{aligned}$$

which are precisely the singularities that appeared in (52). Eventually, one can show that all t-channel gauge field singularities are generated by taking into account the following rule and vertices in the EFT:

$$\begin{aligned}&{\mathcal {A}}=V^{a}(C_{p-2},T_3,A)G^{ab}(A)V^{b}(A,A_1,A_2),\\&V^{a}(C_{p-2},T_3,A)=2\mu '_p\beta '(2\pi \alpha ')^{2}\frac{1}{(p-2)!} \epsilon ^{a_{0}\cdots a_{p-2}ac} p_c \nonumber \\&\quad \times C_{a_{0}\cdots a_{p-3}}k_{a_{p-2}} \sum _{n=-1}^{\infty }b_n(\alpha 'k_3\cdot k)^{n+1} , \\&V^{b}(A,A_1,A_2)=-iT_p(2\pi \alpha ')^{2} [\xi _{1}^{b}(k_1-k)\cdot \xi _{2}\nonumber \\&\quad +\,\xi _{2}^{b}(k-k_2)\cdot \xi _{1}+\xi _{1}\cdot \xi _{2}(k_2-k_1)^{b}] , \\&G^{ab}(A)=\frac{i\delta ^{ab}}{(2\pi \alpha ')^{2}T_p t}. \end{aligned}$$

*k* is the off-shell gauge field’s momentum and $V^{a}(C_{p-2},T_3,A)$ was derived from the corrections to the WZ coupling $C_{p-2}\wedge F\wedge DT$. Notice that the kinetic term of the gauge fields is fixed in DBI action, so one finds that $V^{b}(A,A_1,A_2)$ should not receive any higher-derivative corrections. The tachyon expansion that we talked about is also consistent with effective field theory. This is because we are able to produce all tachyon and massless poles of the string amplitude in the EFT as well.

The expansion has also been checked for various other non-supersymmetric cases, such as all the other three and four point functions (like $CAT, C\phi \phi T$). That is why we believe that the expansion is universal. This might indicate that the tachyon momentum expansion is unique. It would be nice to check it with the higher point functions of non-BPS string amplitudes. The precise form of the solutions for integrals of six point functions is unknown. Given the exact symmetries of the amplitudes and the universal tachyon expansion in [42], we were able to obtain all the singularity structures of the amplitude of a closed string RR and four tachyons. We hope to overcome some other open questions in the near future.

## Footnotes

1 $$\alpha '=2$$ is set.

2 $$x_{ij}=x_i-x_j$$, and $$\alpha '=2$$.

3

$$\begin{aligned} L_3= & {} -{\pi ^{5/2}}\sum _{p,n,m=0}^{\infty }\Bigg (c_n(s'+t+u')^n+c_{n,m}\frac{s'^nu'^m +s'^mu'^n}{(t+s'+u')}\\&+\,f_{p,n,m}(s'+t+u')^p (s'+u')^n(s'u')^{m}\Bigg )\nonumber \\&b_{-1}=1,\,b_0=0,\,b_1=\frac{1}{6}\pi ^2 ,e_{1,0,0}=\frac{1}{6}\pi ^2\\&c_0=0,c_1=\frac{\pi ^2}{3},c_{1,0}=c_{0,1}=0, f_{0,0,1} =4\zeta (3). \end{aligned}$$

## References

[1] Polchinski J (1995). Phys. Rev. Lett..

[2] Witten E (1996). Nucl. Phys. B.

[3] M.R. Douglas. arXiv:hep-th/9512077

[4] Minasian R, Moore GW (1997). JHEP.

[5] Witten E (1998). JHEP.

[6] Myers RC (1999). JHEP.

[7] Nurmagambetov A (2013). JHEP.

[8] Nurmagambetov AJ (2013). Nucl. Phys. B.

[9] de Alwis S (2013). JHEP.

[10] Sen A (2005). Int. J. Mod. Phys. A.

[11] Friedan D, Martinec EJ, Shenker SH (1986). Nucl. Phys. B.

[12] Hatefi E (2013). JHEP.

[13] Aganagic M (1997). Nucl. Phys. B.

[14] Li M (1996). Nucl. Phys. B.

[15] Kraus P, Larsen F (2001). Phys. Rev. D.

[16] Sen A (1999). JHEP.

[17] Bergshoeff EA, de Roo M, de Wit TC, Eyras E, Panda S (2000). JHEP.

[18] Garousi MR, Hatefi E (2008). Nucl. Phys. B.

[19] Kennedy C, Wilkins A (1999). Phys. Lett. B.

[20] A. Sen, Non-BPS states and Branes in string theory. arXiv:hep-th/9904207

[21] De Smet PJ, Raeymaekers J (2000). The Tachyon potential in Witten’s superstring field theory. JHEP.

[22] Hatefi E (2013). Selection rules and RR couplings on non-BPS branes. JHEP.

[23] Liu H, Michelson J (2001). Nucl. Phys. B.

[24] Hatefi E (2012). Phys. Rev. D.

[25] Hatefi E (2013). JCAP.

[26] Hatefi E (2017). JHEP.

[27] Bianchi M, Pradisi G, Sagnotti A (1992). Nucl. Phys. B.

[28] Barreiro LA, Medina R (2014). Nucl. Phys. B.

[29] E. Hatefi, Phys. Lett. B **766**, 153-161 (2017). arXiv:1611.00787

[30] Hatefi E (2016). Phys. Lett. B.

[31] Medina R (2002). JHEP.

[32] Medina R (2009). JHEP.

[33] Medina R (2010). JHEP.

[34] Hatefi E (2015). JHEP.

[35] Hatefi E (2013). JHEP.

[36] Park IY (2009). Eur. Phys. J. C.

[37] Hatefi E, Park IY (2012). Nucl. Phys. B.

[38] Hatefi E, Park IY (2012). Phys. Rev. D.

[39] Fotopoulos A (2001). JHEP.

[40] Hatefi E (2016). Phys. Lett. B.

[41] Gutperle M, Strominger A (2002). JHEP.

[42] Hatefi E (2017). Highly symmetric D-brane–anti-D-brane effective actions. JHEP.

